# Supplementary material for: Using relative brain size as predictor variable: Serious pitfalls and solutions
Source: Ecol Evol. 2022 Sep 20;12(9):e9273. doi: 10.1002/ece3.9273 (PMC9489487; doi:10.1002/ece3.9273)
Supplement: Supplementary file 1 — Figures S1‐S11 [file ECE3-12-e9273-s001.docx]

## Supplemental results

Figure 1 Model with sample size 20. Parameter estimates from the linear model and Bayesian linear model with brain size as response variable. Dashed grey line is the true value. Orange density plots are normal distributions based on the mean and SE from the linear model. Purple density plots are the posterior distributions from the Bayesian models.

Figure 2 Model with sample size 1000. Parameter estimates from the linear model and Bayesian linear model with brain size as response variable. Dashed grey line is the true value. Orange density plots are normal distributions based on the mean and SE from the linear model. Purple density plots are the posterior distributions from the Bayesian models.

Figure 3 Model with sample size 20. Parameter estimates from the linear model, Bayesian linear model and Bayesian structural equation model with relative brain size as predictor variable. Dashed grey line is the true value. Orange density plots are normal distributions based on the mean and SE from the linear model. Purple and green density plots are the posterior distributions from the Bayesian models.

Figure 4 Model with sample size 1000. Parameter estimates from the linear model, Bayesian linear model and Bayesian structural equation model with relative brain size as predictor variable. Dashed grey line is the true value. Orange density plots are normal distributions based on the mean and SE from the linear model. Purple and green density plots are the posterior distributions from the Bayesian models.

Figure 5 Model with informative priors. Parameter estimates from the linear model and Bayesian linear model with brain size as response variable. Dashed grey line is the true value. Orange density plots are normal distributions based on the mean and SE from the linear model. Purple density plots are the posterior distributions from the Bayesian models.

Figure 6 Model with vague priors. Parameter estimates from the linear model and Bayesian linear model with brain size as response variable. Dashed grey line is the true value. Orange density plots are normal distributions based on the mean and SE from the linear model. Purple density plots are the posterior distributions from the Bayesian models.

Figure 7 Model with informative priors. Parameter estimates from the linear model, Bayesian linear model and Bayesian structural equation model with relative brain size as predictor variable. Dashed grey line is the true value. Orange density plots are normal distributions based on the mean and SE from the linear model. Purple and green density plots are the posterior distributions from the Bayesian models.

Figure 8 Model with vague priors. Parameter estimates from the linear model, Bayesian linear model and Bayesian structural equation model with relative brain size as predictor variable. Dashed grey line is the true value. Orange density plots are normal distributions based on the mean and SE from the linear model. Purple and green density plots are the posterior distributions from the Bayesian models.

Figure 9 Model with strong effect of body size and weak effect of brain size. Parameter estimates from the linear model, Bayesian linear model and Bayesian structural equation model with relative brain size as predictor variable. Dashed grey line is the true value. Orange density plots are normal distributions based on the mean and SE from the linear model. Purple and green density plots are the posterior distributions from the Bayesian models.

Figure 10 Model with strong effect of brain size and weak effect of body size. Parameter estimates from the linear model, Bayesian linear model and Bayesian structural equation model with relative brain size as predictor variable. Dashed grey line is the true value. Orange density plots are normal distributions based on the mean and SE from the linear model. Purple and green density plots are the posterior distributions from the Bayesian models.

Figure 11 Model with relative brain size as response variable and an additional causal path from body size to z. Parameter estimates from the linear model and Bayesian linear model. Dashed grey line is the true value. Orange density plots are normal distributions based on the mean and SE from the linear model. Purple density plots are the posterior distributions from the Bayesian model.
